# In Case of Fire

**Published:** 1874-11

**Authors:** 


### IN CASE OF FIRE.

Mr. John Z. Durkee, Fire Marshal of San Francisco, gives the following useful hints to the public as the result of his observation and experience. If these sensible suggestions could be universally read and acted upon, many valuable lives and much property would unquestionably be saved. We can only think of one hint to add to these, and that is this :

When you light a gas burner in a swinging-bracket, see that it does not get pushed back near the wall. We have seen a house set on fire by neglecting this precaution.

1. Be well acquainted with the best means of escape from your house, both at the top and the bottom.

2. Do not get confused ; admit no one to your house except firemen, policemen, or neighbors.

3. If a lady's or a child's dress takes fire, endeavor to roll the person up in a rug, carpet, or any piece of woolen stuff.

4. Keep all doors and windows closed until the firemen arrive.

5. Always keep in your bedroom a piece of rope sufficiently long to reach the sidewalk, in case you cannot make your exit by the stairway.

6. If you cannot make your way from a building by the stairway, endeavor to get in a front room, and be careful to keep all doors shut behind you, for smoke will follow a draft, and flames the smoke. If smoke enters the room, and it is difficult to stand erect, get your mouth as close to the floor as possible, and breathe easy, as there is always a fresh current of air near the floor. A wet cloth over the mouth will greatly aid breathing.

7. In getting smoke from a room, always open the upper portion of a window.

8. In case of a fire in a theatre, or any place where numbers of persons are, keep perfectly cool, and do all you can to prevent a panic, as there is generally plenty of time to escape if there is no panic.

9. Do not go into a building where there is a thick smoke if you can help it, without a saturated sponge in your mouth or a wet cloth or handkerchief over mouth and nose.

10. In ascending or descending a ladder, do so with a regular step, to prevent vibration.

11. Have metal or earthen vessels for matches, and keep them out of the reach of children. Wax matches are not safe.

12. Never leave small children in a room alone where there are matches or an open fire.

13. Do not deposit ashes in a wooden vessel or upon a wooden floor.

14. Never use a light in examining a gas meter.

15. Never take a light into a closet.

16. Never smoke or read in bed by candle or lamp light.

17. Never put kindling wood on the top of a stove to dry.

18. Never leave clothes near a grate or fireplace to dry.

19. Be careful in making fires with shavings, and never use any kind of oil to kindle a fire.

20. Keep all lights as far from curtains as possible.

21. Never pour out liquor near an open light.

22. Always fill and trim your lamps by daylight, and never near a fire.

23. Never blow out a fluid lamp.

24. Never allow fluids used for lamps to be kept in a room where a light or fire is used.

25. Always try your coal oil by pouring a little of it in a saucer or cup, and if you can make it burn with a match or a piece of paper, do not use it.

26. Put wirework over your gaslights in show windows, and keep your goods from them.

27. Benzine, naptha, gasoline, camphene, varnish, turpentine, ethereal oil, &c., should never be drawn by candle or lamp light, or in a room where there is a fire.

28. Sand in place of sawdust should be used on the floors of oil stores, drug stores, &c.

29. Always use a closed lantern, and never allow smoking in hay barns, stables, warehouses, or in stores where goods are closely packed.

30. Always keep shavings and fine kindling wood away from steam boilers and furnaces.

31. Keep lofts, cupboards, corners, boxes, &c., free from greasy rags.

32. Before leaving your place of business, see that all lights and fires are out.

33. Before going to bed see that all your lights and fires are out, and that no ashes have been placed in any wooden vessel.

34. See that your stove pipes enter well in the chimney.

